# Identification of protein complexes from multi-relationship protein interaction networks

**DOI:** 10.1186/s40246-016-0069-z

**Published:** 2016-07-25

**Authors:** Xueyong Li, Jianxin Wang, Bihai Zhao, Fang-Xiang Wu, Yi Pan

**Affiliations:** 1 School of Information Science and Engineering, Central South University, Changsha, 410083 China; 2 Department of Information and Computing Science, Changsha University, Changsha, 410003 China; 3 Department of Mechanical Engineering and Division of Biomedical Engineering, University of Saskatchewan, Saskatoon, SK S7N 5A9 Canada; 4 Department of Computer Science, Georgia State University, Atlanta, GA 30302-4110 USA

## Abstract

**Background:**

Protein complexes play an important role in biological processes. Recent developments in experiments have resulted in the publication of many high-quality, large-scale protein-protein interaction (PPI) datasets, which provide abundant data for computational approaches to the prediction of protein complexes. However, the precision of protein complex prediction still needs to be improved due to the incompletion and noise in PPI networks.

**Results:**

There exist complex and diverse relationships among proteins after integrating multiple sources of biological information. Considering that the influences of different types of interactions are not the same weight for protein complex prediction, we construct a multi-relationship protein interaction network (MPIN) by integrating PPI network topology with gene ontology annotation information. Then, we design a novel algorithm named MINE (identifying protein complexes based on Multi-relationship protein Interaction NEtwork) to predict protein complexes with high cohesion and low coupling from MPIN.

**Conclusions:**

The experiments on yeast data show that MINE outperforms the current methods in terms of both accuracy and statistical significance.

## Background

With the completion of the sequencing of the human genome, proteomic research becomes one of the most important areas in the life science. One important task in proteomics is to detect protein complexes based on protein-protein interaction (PPI) data generated by various experimental technologies, e.g., yeast-two-hybrid [1], tandem affinity purification [2], and mass spectrometry [3]. Protein complexes are molecular aggregations of proteins assembled by PPIs, which play critical roles in biological processes. Many proteins are functional only when they are assembled into a protein complex and interact with other proteins in this complex. Protein complexes are key molecular entities to perform cellular functions. Even in the relatively simple model organism *Saccharomyces cerevisiae*, these complexes are comprised of many subunits that work in a coherent fashion. Besides applications of PPI networks, such as protein function predictions [4] and essential protein discoveries [5–11], prediction of protein complexes is another active topic. Actually, protein complexes are of great importance for understanding the principles of cellular organization and function.

Many computational methods for predicting protein complexes from PPI networks have been developed. Pair-wise protein interactions can be modelled as a graph or network, where vertices are proteins and edges are PPIs. Since proteins in the same complex are highly interactive with each other, protein complexes generally correspond to dense subgraphs in the PPI network and many previous studies have been proposed based on this observation, such as MCODE (Molecular Complex detection) [12], MCL (Markov Cluster algorithm) [13], R-MCL (Regularized MCL) [14], CMC (Maximal Clique algorithm) [15], RRW (Repeated Random Walks) [16], SPICi (Speed and Performance in Clustering algorithm) [17], HC-PIN (Hierarchical Clustering based on Protein-Protein Interaction Network) [18], IPC-MCE (Identifying Protein Complexes based on Maximal Clique Extension) [19], and IPCA (Identification of Protein Complexes Algorithm) [20]. Nepusz et al. [21] proposed an algorithm to find overlapping protein complexes from PPI networks, named ClusterONE (Clustering with Overlapping Neighborhood Expansion). For the convenience of researchers, MCODE, ClusterONE, etc. have been designed as plus-in for protein complex prediction and biological network analysis. ClusterViz [22] is such a Cytoscape APP to complete this work.

However, these abovementioned approaches for extracting dense subgraphs fail to take into account the inherent organization. Recent analysis of experimentally detected protein complexes [23] has revealed that a complex consists of a core component and attachments. Core proteins are highly co-expressed and share high functional similarity, and each attachment protein binds to a subset of core proteins to form a biological complex. Based on the core-attachment concept, some algorithms have been proposed, including COACH (Core-Attachment-based method) [24], CORE [25], MCL-Caw [26], DCU (Detecting Complex based on Uncertain graph model) [27], and WPNCA (a Weighted PageRank-Nibble algorithm with Core-Attachment structure) [28].

In spite of the advances in computational approaches and related fields, accurate identification protein complexes are still a bottleneck. One of the most important reasons is that the PPI network contains a lot of false positives which greatly reduce the complex detection accuracy. To address this problem, biological information other than PPIs has been integrated with network topology to improve the precision of protein complex detection methods. Wu et al. proposed a method called CACHET to discover protein complexes with core-attachment structures from tandem affinity purification (TAP) data [29]. Tang et al. [30] constructed time course PPI networks by incorporating gene expression into PPI networks and applied it successfully to the identification of function modules. Wang et al. [31] proposed a three-sigma method to identify active time points of each protein in a cellular cycle, where three-sigma principle is used to compute an active threshold for each gene according to the characteristics of its expression curve. A dynamic PPI network (DPIN) is constructed for the detection of protein complexes. Li et al. proposed novel algorithms, such as TSN-PCD [32] and DPC [33], to identify dynamic protein complexes by integrating PPI data and dynamic gene expression profiles. Zhao et al. [34] reconstructed a weighted PPI network by using dynamic gene expression data and developed a novel protein complex identification algorithm, named PCIA-GeCo.

There exist complex and diverse relationships among proteins after integrating multiple sources of biological information. However, comparing PPI data is difficult because they are often diverse and play different roles under different conditions. Current existing approaches failed to take into account and combined the interactions with different natures into one interaction effectively. Taking into account the influences of different types of interactions are not the same weight for protein complex prediction, we construct a multi-relationship protein interaction network (MPIN) by integrating PPI network topology with gene ontology (GO) annotation information. Then, a new method named MINE (identify protein complexes based on Multi-relationship protein Interaction NEtwork) is proposed. We have conducted an experiment on yeast data. Experimental results show that MINE outperforms the existing methods in terms of both accuracy and *p* value.

## Methods

### Multi-relationship protein interaction network

Complex networks have now been a new research focus because of surging networks in various fields such as engineering, social science, and life science. In reality, connections among nodes in complex networks are diversified. Multi-relationship means that there is more than one connection between two nodes and each of them has its own property. For instance, in social networks [35], persons contact with each other via emails, telephones, MSN, etc. and hence make up a complex multi-relationship network. Similarly, in biological networks, there are diverse links among proteins like physical interaction, co-expression, and co-annotation. However, multi-relationship networks are much more difficult to analyze than single-relationship networks. Multi-relationship networks are also essential in better reflecting the real world.

#### Definition 1 *Multi-relationship network*

Consider a PPI network *G* = (*V*, *E*), where *V* = {*v*_1_, *v*_2_,…, *v*_*n*_} represents a set of proteins and *E* = {*e*_1_, *e*_2_,…, *e*_*m*_} represents a set of interactions. A multi-relationship network is defined as MG = (*V*, *E*∪*E*’, *T*), where *T*(*e*_*i*_) = *t*_*i*_ (*i* = 1, 2…*m*) is the interaction type of *e*_*i*_. *E*’ is the set of new generated interactions.

**Fig. 1:**
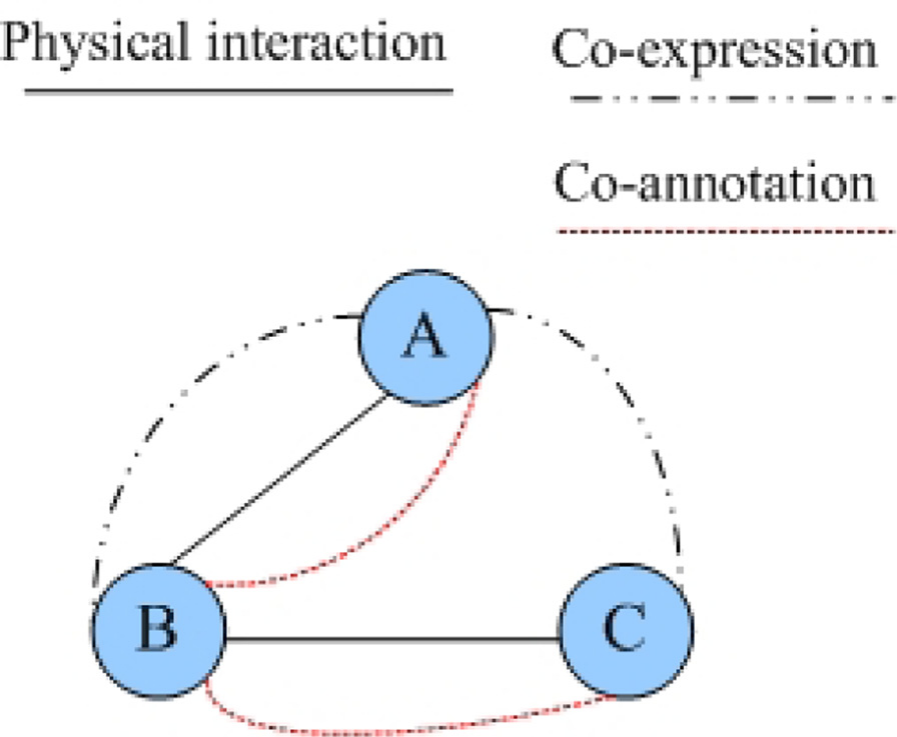
An example of a typical multi-relationship network. There are three links between A and B, including physical interaction, co-expression, and co-annotation. *Solid line* represents physical interaction of PPI networks, *dotted line* represents co-annotation between two nodes, and the *rest line* indicates co-expression

In a multi-relationship network, a pair of proteins may be connected by more than one type of links. If there are two or more links between a pair of proteins, they are called parallel interactions. Figure 1 illustrates a typical multi-relationship network. From Fig. 1, we can see that proteins A and B have physical interaction in the PPI network and at the same time, A and B are also co-expression based on gene expression profiles and co-annotations based on gene ontology annotation information. In the multi-relationship network, multiple connections between A and B are kept.

Researches [27, 36] show that PPI data obtained through high-throughput biological experiments contains relatively high rates of false positives and false negatives. False positives become obstacle to the precision of prediction algorithm. False negatives lead to the loss of interaction data and continue to inhibit the increase of the number of protein complexes correctly matched. To overcome these problems, researches have begun to integrate the PPI network and other biological information, such as gene expression profiles, essential proteins, and GO annotation information. Due to the similar biological properties of protein complexes, GO annotation is a valuable addition to PPI data for protein complex prediction. Therefore, in this study we construct a multi-relationship protein interaction network by integrating PPI network topology and GO annotation information.

**Fig. 2:**
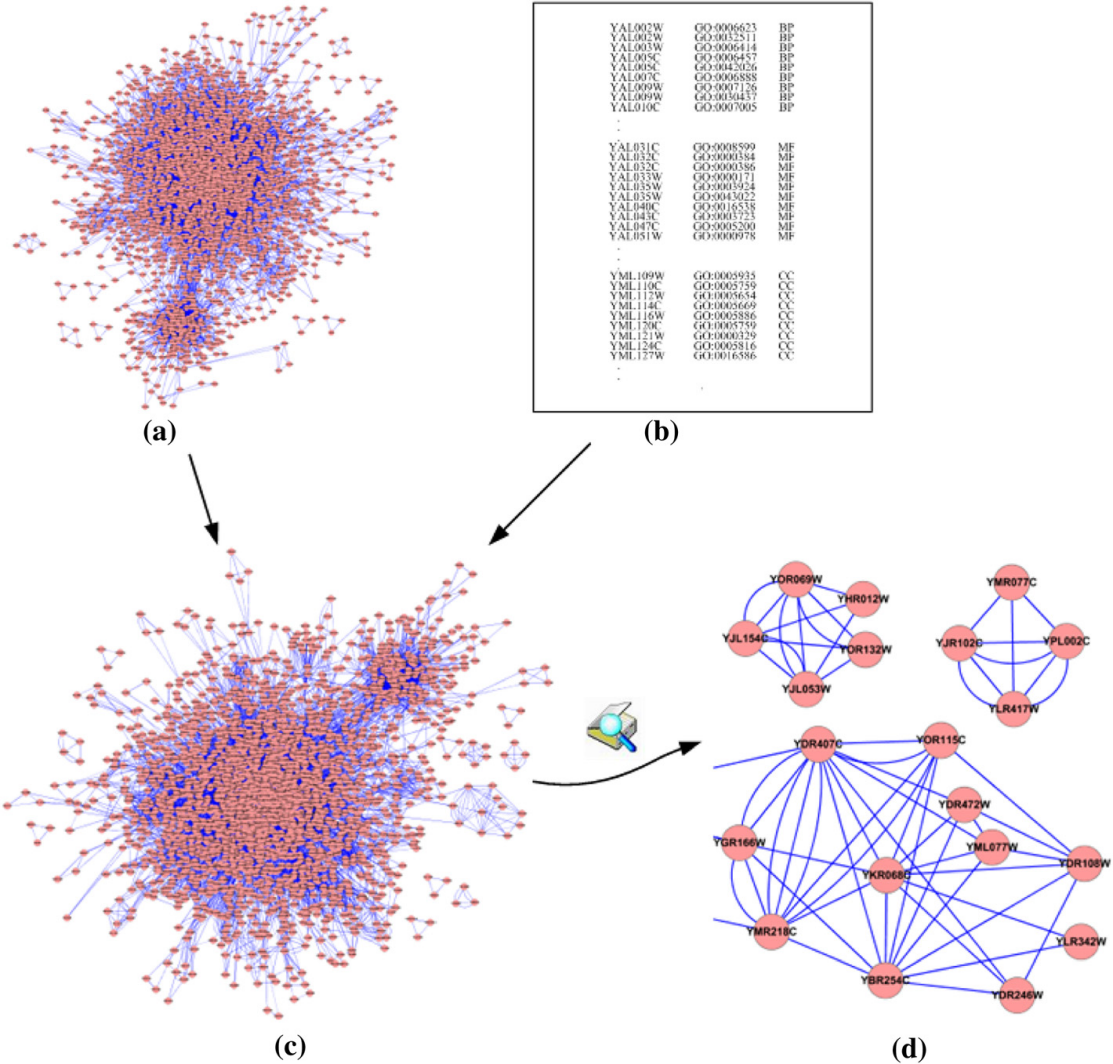
Schematic of construction of a multi-relationship protein interaction network. After inputting a PPI network and GO annotation files, MINE algorithms output a weighted multi-relationship protein interaction network. **a** The original PPI network. **b** The GO annotation file, including BP, MF, and CC. **c** The constructed multi-relationship protein interaction network by integrating PPI networks and GO annotation. **d** An example of a magnified sub-network of the multi-relationship network

The GO database consists of three separate categories of annotations, namely molecular function (MF), biological process (BP), and cellular component (CC). MF describes activities, such as catalytic or binding activities, at the molecular level. BP describes biological goals accomplished by one or more ordered assemblies of molecular functions. CC describes locations, at the levels of subcellular structures and macromolecular complexes. In this study we integrate the PPI network and three categories of GO annotations to construct a multi-relationship protein interaction network. In our constructed multi-relationship network, four kinds of interactions at most can be considered between two proteins, namely the interactions of the PPI network and the interactions of sharing molecular functions, sharing biological processes, and sharing cellular components. Figure 2 describes the process of a multi-relationship network construction.

In the constructed multi-relationship protein interaction network, two proteins are connected if they interact with each other in the PPI network or have common functions, including biological processes, molecular functions, and cellular components. After constructing a multi-relationship protein interaction network, we do some further processing, such as weighting and filtering. Studies [9, 10, 36] show that the performance of prediction algorithms based on weighted networks is generally superior to that based on un-weighted networks. The reason is simple: weight stands for the relative reliability/importance of interactions; thus, weighted networks can be more valuable than un-weighted networks in the representative of PPI networks. For the first type of interaction in our constructed multi-relationship network, interacting with each other in the PPI network, we weight these interactions through the analysis of topological features of PPI networks. Generally speaking, for a pair of interacting proteins, the strength of an interaction can be reflected by the number of its common neighbors. This study uses ECC to calculate the weight of protein pairs, which is defined as

1 $$ \mathrm{E}\mathrm{C}\mathrm{C}\left({v}_i,{v}_j\right)=\left\{\begin{array}{ccc}\hfill \frac{\left|{N}_i\cap {N}_j\right|{}^2}{\left(\left|{N}_i\right|-1\right)*\left(\left|{N}_j\right|-1\right)}\hfill & \hfill, \hfill & \hfill \left|{N}_i\right|>1\ \mathrm{and}\ \left|{N}_j\right|>1\hfill \\ {}\hfill 0\hfill & \hfill, \hfill & \hfill \left|{N}_i\right|=1\ \mathrm{or}\ \left|{N}_j\right|=1\hfill \end{array}\right., $$

where *N*_*i*_ and *N*_*j*_ are the neighborhood sets of *v*_*i*_ and *v*_*j*_, respectively. To reduce the negative effect of false positive on the protein complex prediction, we remove interactions whose ECC values are zero.

For the rest three types of interaction, we weight interactions according to the number of common functions (including BP, MF, and CC) between two proteins. For a pair of proteins *v*_*i*_ and *v*_*j*_, BP_*i*_ and BP_*j*_ are sets of biological processes of *v*_*i*_ and *v*_*j*_, respectively. W_BP (*v*_*i*_, *v*_*j*_) represents the strength of sharing biological processes, which is calculated as follows:

2 $$ \mathrm{W}\_\mathrm{B}\mathrm{P}\left({v}_i,{v}_j\right)=\left\{\begin{array}{ccc}\hfill \frac{\left|{\mathrm{BP}}_i\cap {\mathrm{BP}}_j\right|{}^2}{\left|{\mathrm{BP}}_i\left|*\right|{\mathrm{BP}}_j\right|}\hfill & \hfill, \hfill & \hfill \left|{\mathrm{BP}}_i\right|*\left|{\mathrm{BP}}_j\right|>0\hfill \\ {}\hfill 0\hfill & \hfill, \hfill & \hfill \left|{\mathrm{BP}}_i\right|*\left|{\mathrm{BP}}_j\right|=0\hfill \end{array}\right. $$

In Eq. (2), BP_*i*_∩BP_*j*_ denotes the set of common biological processes of *v*_*i*_ and *v*_*j*_. In a similar way, W_MF (*v*_*i*_, *v*_*j*_) and W_CC (*v*_*i*_, *v*_*j*_) denote the strengths of sharing molecular functions and cellular components of *v*_*i*_ and *v*_*j*_, respectively. They can be calculated as follows:

3 $$ \mathrm{W}\_\mathrm{M}\mathrm{F}\left({v}_i,{v}_j\right)=\left\{\begin{array}{ccc}\hfill \frac{\left|{\mathrm{MF}}_i\cap {\mathrm{MF}}_j\right|{}^2}{\left|{\mathrm{MF}}_i\left|*\right|{\mathrm{MF}}_j\right|}\hfill & \hfill, \hfill & \hfill \left|{\mathrm{MF}}_i\right|*\left|{\mathrm{MF}}_j\right|>0\hfill \\ {}\hfill 0\hfill & \hfill, \hfill & \hfill \left|{\mathrm{MF}}_i\right|*\left|{\mathrm{MF}}_j\right|=0\hfill \end{array}\right. $$

4 $$ \mathrm{W}\_\mathrm{C}\mathrm{C}\left({v}_i,{v}_j\right)=\left\{\begin{array}{ccc}\hfill \frac{\left|{\mathrm{CC}}_i\cap {\mathrm{CC}}_j\right|{}^2}{\left|{\mathrm{CC}}_i\left|*\right|{\mathrm{CC}}_j\right|}\hfill & \hfill, \hfill & \hfill \left|{\mathrm{CC}}_i\right|*\left|{\mathrm{CC}}_j\right|>0\hfill \\ {}\hfill 0\hfill & \hfill, \hfill & \hfill \left|{\mathrm{CC}}_i\right|*\left|{\mathrm{CC}}_j\right|=0\hfill \end{array}\right. $$

For the three types of interactions, we perform more stringent filter operations than the first type because they are newly generated interactions. For a pair of function-shared proteins, if they have only one common function or no common neighbors in the PPI network, interactions between them are removed. After performing the above operations, a weighted multi-relationship protein interaction network is constructed.

### MINE algorithm

**Fig. 3:**
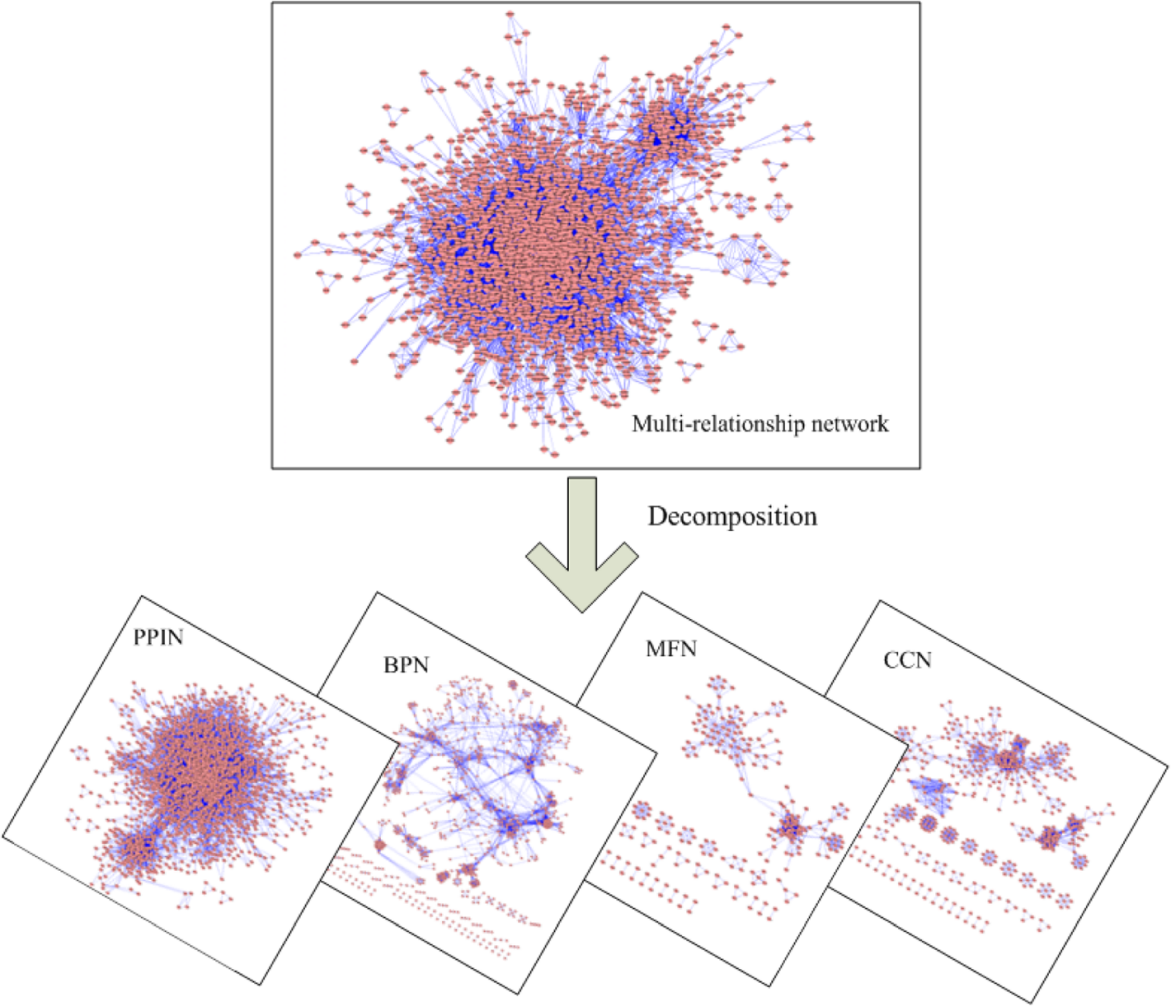
Decomposition of a multi-relationship protein interaction network. The multi-relationship network is broken into several single networks, including PPI network, BPN (sharing biological process network), MFN (sharing molecular function), and CCN (sharing cellular component)

Considering the influences of different types of interactions in protein complex prediction are not the same, we construct a multi-relationship protein interaction network by integrating PPI networks and GO annotation information. To test the effectiveness of the multi-relationship network, we design a new method for predicting protein complexes, named MINE (based on Multi-relationship protein Interaction NEtwork). Multi-relationship networks have more complex attributes than single networks. Current protein complex prediction methods are mainly based on single networks. So, converting a multi-relationship network into single networks is key to design the MINE algorithm. A simple way for addressing this problem is to combine interactions with different natures to one interaction effectively. In reality, it is inappropriate for us to combine multiple interactions between two proteins because they are often derived under different conditions and play different roles in protein complex prediction. Considering that different types of interactions play different roles in detecting protein complexes, we decompose the multi-relationship network into several single networks, including the PPI network, BPN (sharing biological processes), MFN (sharing molecular functions) and CCN (sharing cellular components). Figure 3 displays the framework of multi-relationship decomposition.

And then, we identify protein complexes through mining density subgraphs from the four networks. Intuitively, a subgraph representing a protein complex should satisfy two simple structural properties: it should contain many reliable interactions between its subunits, and it should be well-separated from the rest of the network [21]. Inspired by the notion, we take into account the density of a subgraph and connections between nodes of the subgraph and nodes out of the subgraph. To describe MINE simply and clearly, we provide the following definitions, firstly.

#### Definition 2 *Weighted Density* [27]

Given a weighted network *G* = (*V*, *E*, *W*). *V* = {*v*_1_, *v*_2_, …, *v*_*n*_}, *E* = {*e*_1_, *e*_2_,…, *e*_*m*_}, *W* = {*w*(*e*_1_), *w*(*e*_2_),…, *w*(*e*_*m*_)}, *w*(*e*_i_) is the weight of an edge *e*_i_. WD (*G*) denotes the weighted density of *G* and is defined as

5 $$ \mathrm{W}\mathrm{D}(G)=\frac{{\displaystyle \sum_{i=1}^mp\left({e}_i\right)\times 2}}{\underset{1\le i\le \Big|m}{ \max}\left(p\left({e}_i\right)\right)\times \left(\left|V\right|\times \left(\left|V\right|-1\right)\right)} $$

#### Definition 3 *Sub-network Weighted Degree* [36]

Given a weighted sub-network *G* = (*V*, *E*, *W*) and a vertex *u*, *u*∊*V. V* = {*v*_1_, *v*_2_, …, *v*_*n*_}, *E* = {*e*_1_, *e*_2_,…, *e*_*m*_}, *W* = {*w*(*e*_1_), *w*(*e*_2_),…, *w*(*e*_*m*_)}, *w*(*e*_i_) is the weight of an edge *e*_i_. SWD (*u*, *G*) denotes the weighted degree of *u* within *G* and is defined as

6 $$ \mathrm{S}\mathrm{W}\mathrm{D}\left(u,G\right) = {\displaystyle \sum_{i=1}^nw\left(u,{v}_i\right)},\left(u,{v}_i\right)\in E $$

Based on these definitions, we are now ready to describe our proposed MINE algorithm to detect protein complexes. Our method visits the four single networks, respectively, to discover density subgraphs as protein complexes. For a selected network, MINE starts from a randomly chosen protein vertex and add protein vertices via a greedy procedure to form a candidate complex with high cohesion and low coupling. The growth process is repeated from all vertices to form non-redundant complex sets. Since some vertices have similar neighborhood graphs, the candidate complexes detected from their neighborhood graphs may have large overlaps, which result in high redundancy. Hence, a redundancy-filtering procedure is applied to quantify the extent overlap between each pair of complexes and discard the complexes with low density or small size.

**Fig. 4:**
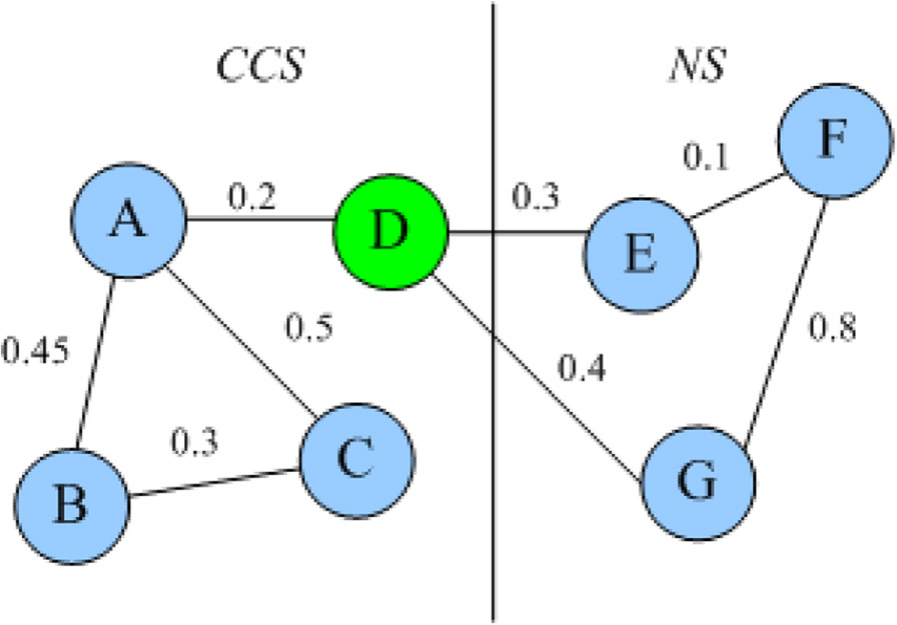
An example of removing high-coupling proteins. The sum of weighted degree in CCS is 0.2, while that value in NS is 0.7, so *D* is removed from CCS, due to high coupling with neighbor set NS

MINE algorithm (Algorithm 1) describes the overall procedure to identify protein complexes. MINE algorithm processes four single networks according to the multi-relationship network, such as PPIN, BPN, MFN, and CCN, in line 1. For a selected network *G*_*k*_, we first generate candidate complexes according to neighbors of all proteins in the network, in lines 3–8. The seed is inserted into the candidate set CCS, and then all neighbors of the seed are put into CCS one by one. If the weighted density of CCS is less than the threshold WDT, the new added neighbor node is removed from CCS. After this process, a candidate complex with high cohesion is formed. Then, we remove some nodes highly connected with the neighbor subgraph to form a candidate complex with low coupling, in lines 9–12. Figure 4 illustrates an example of removing high-coupling proteins. In Fig. 4, SWD(*D*, CCS) = 0.2, SWD(*D*, NS) = 0.3 + 0.4 = 0.7, *D* is removed from CCS.

Finally, if CCS is not a subset of complex in the set of protein complex SC, CCS is inserted into SC.

The second stage of our method is redundancy-filtering, in lines 15–20. Complexes overlapping to a very high extent should be discarded. With quantifying the extent of overlap between each pair of complexes, a complex with small weighted density or a small number of proteins is discarded for which overlap score of the pair is above the threshold. In our method, the overlap threshold is typically set as 0.8 [21, 27], where the matching score of two complexes *A* and *B* is defined as follows [15, 24]:

7 $$ \mathrm{M}\mathrm{S}\left(A,B\right)=\frac{\left|A\cap B\right|{}^2}{\left|A\left|\times \right|B\right|} $$

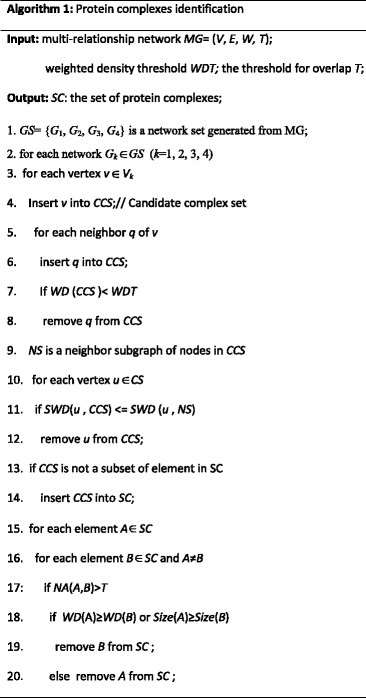


## Results and discussion

In order to evaluate the performance of our proposed algorithm, we compare it with other five competing algorithms, including CMC [15], RRW [16], COACH [24], SPICi [17], and ClusterONE [21]. For all those competing algorithms, the parameters are set as recommended by their authors. We have applied our MINE method and other methods on two yeast PPI networks, including DIP [37] and Krogan [38]. These PPI datasets are available online, which varied from each other a lot. In this section, we will first present in details the results on DIP data. The results using Krogan data will also be briefly presented to demonstrate the effectiveness of our proposed method.

The DIP dataset consists of 5023 proteins and 22,570 interactions. The Krogan dataset contains 3672 proteins and 14,317 interactions. Self-interactions and repeated interactions are filtered out in the three PPI networks. To evaluate the protein complexes predicted by our method, a benchmark set is obtained from the reference [39], which consists of 408 complexes.

To assess the quality of predicted complexes, we employed several evaluation measures, including precision, recall, *F*-measure, and functional enrichment of GO terms.

### Precision, recall, and *F*-measure

We describe how well the predicted protein complexes match with the benchmark complex set, firstly. A predicted protein complex is considered to match with a benchmark complex, if its matching score MS (see Eq. (7)) is no less than a threshold. Typically, the threshold is set as 0.2 [24, 27]. Precision and recall are the commonly used measures to evaluate the performance of protein complex prediction algorithms. Precision measures the percentage of predicted protein complexes that match benchmark complexes in all the predicted protein complexes. Recall is the fraction of benchmark complexes that are retrieved. Mathematically, precision and recall are defined as follows:

8 $$ \mathrm{Precision}=\frac{N_{\mathrm{cp}}}{\left|P\right|} $$

9 $$ \mathrm{Recall}=\frac{N_{\mathrm{cb}}}{\left|B\right|} $$

where *N*_cp_ is the number of predicted complexes matched by benchmark complexes, *N*_cb_ is the number of benchmark complexes that are matched by predicted complexes, *P* is the set of predicted protein complexes and *B* is the benchmark complex set.

*F*-measure, as the harmonic mean of precision and recall, can be used to evaluate the overall performance of the different techniques [21, 24]. Table 1 shows the basic information about predicted complexes by various methods on DIP data, where the best values are italized.

**Table 1:** The matching results of various algorithms

Algorithms	PC	*N*_cp_	*N*_cb_	*N*_pcp_
MINE	*606*	*345*	*218*	*19*
CMC	235	119	124	8
COACH	902	319	219	15
RRW	250	118	136	4
SPICi	574	118	143	7
ClusterONE	371	155	136	6

**Fig. 5:**
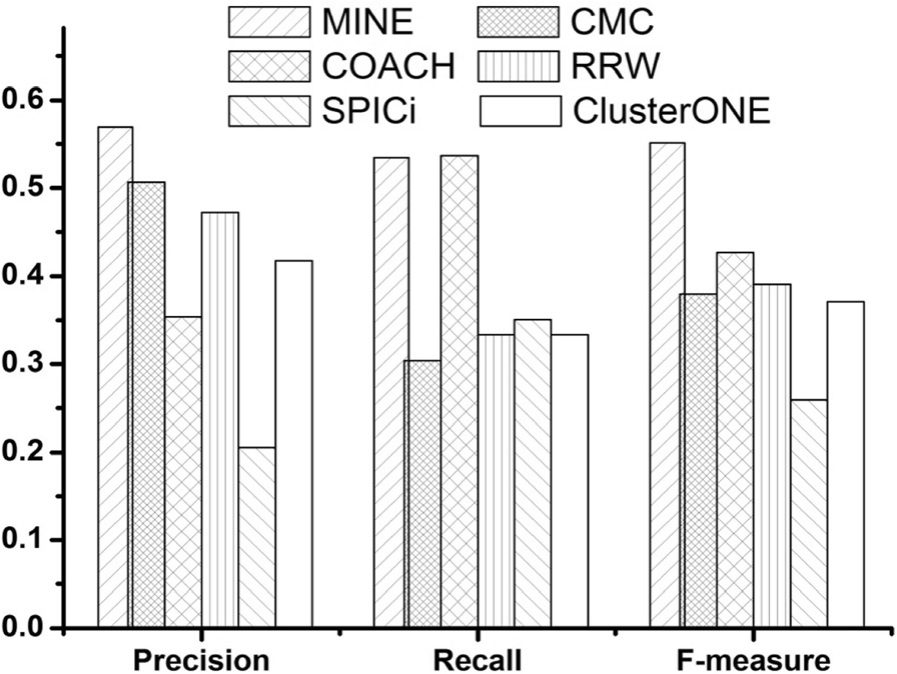
The performance comparison for various algorithms on DIP data. MINE achieves the highest precision, recall, and *F*-measure among all the six methods

In Table 1, PC represents the total number of predicted complexes, while *N*_pcp_ is the number of complexes perfectly matching the benchmark complexes. In other words, the matching score between a predicted complex and a benchmark complex is 1. From Table 1, we can see that MINE produces the largest number of correctly predicted complexes and the second-largest number of benchmark complexes after COACH, respectively, while PC of our method (606) is far less than COACH’s (902). The fifth column of Table 1 shows that MINE has the absolute advantage to obtain the largest number of perfectly matched complexes. *N*_pcp_ of MINE is 137.5, 26.67, 375, 171.43, and 216.67 % higher than that of CMC, COACH, RRW, SPICi, and ClusterONE, respectively. Figure 5 shows the overall comparison in terms of precision, recall, and *F*-measure.

On DIP data, *F*-measure of MINE is 0.551, which is 45.05, 29.23, 41.02, 112.62, and 48.59 % higher than that of CMC, COACH, RRW, SPICi, and ClusterONE, respectively. Our MINE method can achieve the highest *F*-measure by providing the highest precision and the same highest recall as COACH, which shows that our method can predict protein complexes very good.

### Functional enrichment analysis

Another evaluation measure is the function enrichment which measures the biological significance of predicted protein complexes by various algorithms. To substantiate the biological significance of our predicted complexes, we calculate their *p* values, which represent the probability of co-occurrence of proteins with common functions [27]. In this wok, we employ the tool BiNGO [40] to calculate *p* values for predicted complexes. BiNGO is a Java-based tool to determine which GO categories are statistically overrepresented in a set of genes or a subgraph of a biological network. BiNGO is implemented as a plug-in for Cytoscape [41], which is an open-source bioinformatics software platform for visualizing and integrating molecular interaction networks. A low *p* value of a predicted complex indicates that those proteins in the complex do not happen merely by chance, so the complex has high statistical significance. Generally, a complex is considered to be significant with *p* value <0.01. In addition, the *p*-score is also used as an effective evaluation measure, which is defined as

10 $$ p\hbox{-} \mathrm{score}=\frac{1}{n}{\displaystyle \sum_{i=1}^n- \lg \Big(p\kern0.5em {\mathrm{value}}_i}\left)\right|p\kern0.5em {\mathrm{value}}_i<0.01 $$

Table 2 lists comparative results of various algorithms based on GO annotation, where the best values are italized. In Table 2, SC is the number of significant predicted complexes. That is, their *p* values are less than 0.01. Our MINE method achieves the highest proportion of significantly predicted complexes and *p*-score values among all algorithms. The *p*-score of MINE is 12.16, 18.41, 32.08, 48.38, and 20.20 % higher than that of CMC, COACH, RRW, SPICi, and ClusterONE, respectively. In addition, Table 2 indicates that RRW gets the highest proportion of significant complexes, while achieves a lower *p*-score values than ClusterONE because the *p* value of significant complexes predicted by ClusterONE are lower than RRW’s. These results suggest that the complexes predicted by MINE had the most biological significance.

**Table 2:** The comparison of various methods in terms of function enrichment

Algorithms	PC	SC	Proportion (%)	*p*-score
MINE	*606*	*499*	*82.34*	*11.9*
CMC	235	187	79.57	10.61
COACH	902	676	74.94	10.05
RRW	250	191	76.40	9.01
SPICi	574	262	45.64	8.02
ClusterONE	371	235	63.34	9.9

### Effect of parameters on prediction performance

**Fig. 6:**
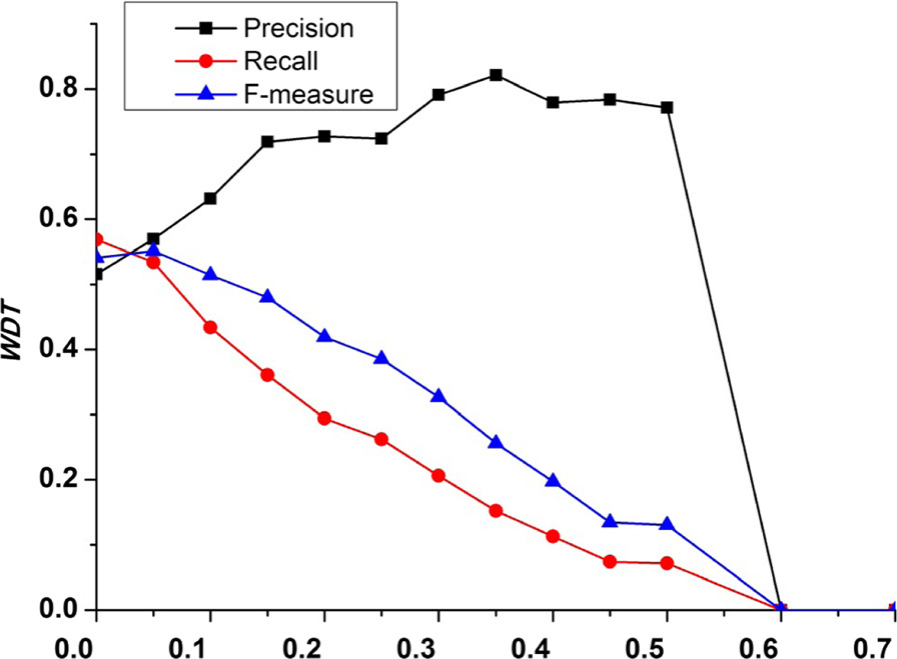
The effect of threshold WDT. It shows that the precision, recall, and *F*-measure of our method fluctuate under various values of WDT. MINE gets the best overall performance when WDT is assigned as 0.05

In MINE, we introduce a user-defined parameter WDT (weighted density threshold) to discover density subgraphs with high cohesion to form candidate complexes. To investigate the effect of parameter WDT on performance of MINE, we evaluate the prediction accuracy in terms of precision, recall, and *F*-measure by setting different values of WDT, ranging from 0 to 1. Figure 6 shows that the performance of our method fluctuates under various values of WDT. Figure 6 clearly indicates that MINE gets the best performance when WDT is assigned as 0.05.

### Results using Krogan data

**Fig. 7:**
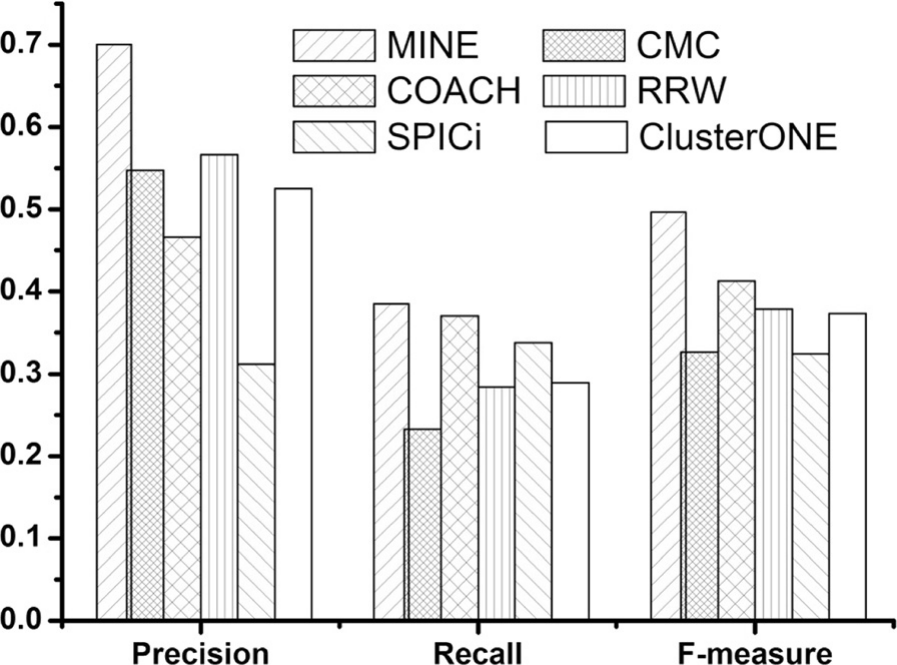
Precision, recall, and *F*-measure of various methods using Krogan data. It shows the performance comparison for the six methods using Krogan data. MINE still archives the best performance among all these methods in terms of precision, recall, and *F*-measure

We also performed MINE method on the Krogan PPI network. The precision, recall, and *F*-measure of each algorithm based on Krogan data are shown in Fig. 7.

Figure 7 indicates that our method gets the best performance among all these methods in terms of precision, recall, and *F*-measure. The *F*-measure of our method is 0.5, which is 68.63, 33.52, 45.53, 69.71, and 47.73 % higher than that of CMC, COACH, RRW, SPICi, and ClusterONE, respectively.

## Conclusions

In this paper, we have constructed a multi-relationship protein interaction network (MPIN) by integrating PPI network topology with GO annotation information. For a pair of proteins in the MPIN, there exists more than one kind of interactions between them. To test the effectiveness of the MPIN, we have developed a novel method named MINE to predict protein complexes. MINE first decomposes the MPIN into four single relationship networks. Then, MINE visits four networks in turn for predicting protein complexes with high cohesion and low coupling. The results of experiments based on yeast PPI networks show that not only MINE achieves higher prediction accuracy than other existing methods but also majority of complexes predicted by MINE possess high biological significance. All results have proved that the constructed MPIN is useful for predicting protein complexes.
